# An Optimal Spray Device for the Nose-to-Brain Delivery of AmyP53, an Adaptive Therapeutic Peptide for Alzheimer’s and Parkinson’s Diseases

**DOI:** 10.3390/pharmaceutics18080987

**Published:** 2026-08-10

**Authors:** Gonçalo Farias, Henri Chahinian, Nathalie Hauchard, Dominique Brunet, Jacques Fantini, Nouara Yahi, Driss Fantini, Anaïs Aulas

**Affiliations:** 1 Aptar Pharma, 27100 Le Vaudreuil, France; goncalo.farias01@aptar.com (G.F.); nathalie.hauchard@aptar.com (N.H.); dominique.brunet@aptar.com (D.B.); 2 Department of Biology, Faculty of Medicine, Aix-Marseille University, 13015 Marseille, France; henri.chahinian@gmail.com (H.C.); jacques.fantini@univ-amu.fr (J.F.); nouara.yahi@univ-amu.fr (N.Y.); 3 AmyPore, 13240 Septèmes-les-Vallons, France; amyp53-project-partner-rfp@amypore.com

**Keywords:** AmyP53, nose to brain, Alzheimer, Parkinson, oligomers, ganglioside, therapy

## Abstract

**Background**: Nose-to-brain delivery offers a noninvasive route to bypass the blood–brain barrier for the treatment of neurodegenerative diseases. AmyP53 is a first-in-class adaptive 12-mer peptide that prevents the formation of neurotoxic amyloid oligomers by competitively targeting lipid raft gangliosides on brain cell membranes, thereby blocking the shared pathological mechanism underlying both Alzheimer’s and Parkinson’s diseases. **Objective**: Here, we report the identification of optimal spray devices for the nose-to-brain delivery of AmyP53, ahead of a planned Phase 1 clinical trial. **Method/Results**: Among six devices evaluated (four commercial systems and two novel devices specifically engineered for nose-to-brain delivery), two systems were identified as optimal for further clinical development (narrower plume angles and significantly higher deposition in the olfactory region): the Neurospray™ and Neurospray™ Preservative-Free (PF). AmyP53 was quantitatively and reproducibly delivered by both Neurospray™ systems, retaining full recognition of its therapeutic target (gangliosides), as assessed by a surface pressure-based ganglioside-binding assay. In a rabbit preclinical model, intranasal administration of AmyP53 with the Neurospray™ resulted in rapid and sustained brain delivery, detectable at 10 min and persisting at 24 h post-administration, without significant systemic exposure. **Conclusions**: These results validate the Neurospray™ drug delivery systems as optimal drug delivery systems for the clinical development of AmyP53.

## 1. Introduction

Nose-to-brain delivery of therapeutic drugs has emerged as a promising approach for the treatment of neurodegenerative diseases, with the key advantage of offering a direct pathway to reach the brain with limited side effects [1,2,3]. The clinical significance of treating central nervous system diseases via the nasal route is evidenced by recent regulatory approvals of several nasal products. Since 2019, the Food and Drug Administration has approved several nasal formulations for epileptic seizures [4,5], depression [6], and migraine [7,8]. Even if none of them claims direct nose-to-brain delivery, their general acceptance due to their efficacy and minor side effects, and the literature around this pathway, suggest that the nose-to-brain route might play a role in drug delivery. As a matter of fact, the nose-to-brain delivery pathway has several advantages over conventional drug administration routes [9]. It also avoids the first-pass metabolism in the liver, allowing for higher bioavailability of therapeutics in the brain at low drug amounts [10,11]. It also provides a faster onset of action while reducing systemic side effects compared to oral or intravenous routes, generally within minutes [12].

The nose-to-brain pathway is thus particularly promising for treating Alzheimer’s and Parkinson’s diseases, with some drugs having already reached phase 2 clinical studies [2,11,13,14,15,16,17,18]. In this regard, we investigated here whether AmyP53, a short synthetic adaptive peptide specifically designed to prevent the formation of neurotoxic amyloid oligomers involved in the pathogenesis of Alzheimer’s and Parkinson’s diseases [19], could benefit from this mode of administration for a Phase I clinical trial in humans. AmyP53 represents the first therapeutic solution targeting a shared pathological mechanism in these two major neurodegenerative disorders: the interaction of amyloid proteins (Aβ in Alzheimer’s, α-Synuclein in Parkinson’s) with brain gangliosides, which leads to the formation of Ca^2+^-permeable amyloid oligomeric pores [20]. Therefore, AmyP53 blocks the calcium influx induced by Aβ and α-Synuclein oligomers and all downstream neurodegenerative processes, including tau hyperphosphorylation, oxidative stress and synaptic dysfunction [20,21,22,23,24]. Regulatory preclinical studies demonstrated both the safety (lack of side effects) and efficient biodistribution of AmyP53 in brain tissues in the minutes following the nose-to-brain administration of AmyP53 in rodents [21,25].

In the present study, we first developed and validated a nasal spray device for the efficient delivery of AmyP53 via the nose-to-brain route in preparation for our future Phase 1 clinical trial. Using the nasal cast, a 3D-printed model of the nasal cavity, we determined which device targeted the zone of interest most efficiently. Second, we confirmed the choice of the administration route using rabbits, an animal model with a similar ganglioside repertoire to that of humans (AmyP53’s targets) [26,27], which is also widely used and well suited for the evaluation of intranasal drug administration in accordance with EMA guidelines.

## 2. Materials and Methods

### 2.1. Four Conventional Commercial Devices Were Compared with Two Novel Drug Delivery Systems Specifically Developed for Nose-to-Brain Delivery

Four conventional commercial devices were compared with two novel drug delivery systems specifically developed for nose-to-brain delivery (the Neurospray™ and Neurospray™-Preservative-Free (PF)). The commercial devices were selected to provide a representative benchmark of the conventional multidose nasal spray technologies currently available on the market, covering different nasal pump technologies and intended applications ranging from local treatments (e.g., allergic rhinitis and vaccination) to systemic drug delivery. These drug delivery systems were originally developed to distribute the formulation broadly across the nasal cavity, without targeting a specific region. All devices tested emitted a volume of approximately 100 µL and were fitted onto white high-density polyethylene bottles filled with different liquids: ultrapure water prepared by a Milli-Q reverse osmosis system (Merck, Darmstadt, Germany), an aqueous 75 mg/mL fluorescein sodium solution (Merck, Darmstadt, Germany), and a 100 mg/mL AmyP53 solution mixed with fluorescein sodium for a final fluorescein sodium concentration of 0.7 mg/mL. The water-filled devices were used to characterize the spray performance, while the addition of fluorescein to the formulation allowed for quantification in the nasal cast without altering the viscosity or spray angle . All device components were supplied by Aptar Pharma (Le Vaudreuil, France).

### 2.2. The Spray Performance Was Characterized by Evaluating the Spray Droplet Size Distribution and Spray Angle

The spray performance was characterized by evaluating the spray droplet size distribution and spray angle, as these parameters are expected to have a significant impact on regional deposition within the nasal cavity [28,29]. The droplet size was measured using a Spraytec^®^ (Malvern Panalytical, Worcestershire, UK) equipped with a 300 mm lens at 4 cm from the laser. The %< 10 µm, Dv10, Dv50 and Dv90 were determined during the stable phase of each product. The spray angle was determined by using a SprayVIEW^®^ (Proveris Scientific, Hudson, NY, USA) by performing spray pattern testing at 5 cm from the laser and the angle calculated from the average diameter. Both tests were conducted with a Proveris Vereo^®^ actuator at a velocity corresponding to human use for each pump/actuator combination filled with water: 70 mm/s for commercial device 1, commercial device 4, the Neurospray™ and the Neurospray™ PF; 55 mm/s for commercial device 2; and 45 mm/s for commercial device 3. Since all devices used are multidose pumps, an initial characterization of the stroke length was performed by applying a contact force of 0.3 kg and a final force of at least 6.0 kg. The defined stroke length was then used for all spray testing. Ten devices per configuration were tested.

### 2.3. Deposition of Liquid Nasal Sprays

Deposition of liquid nasal sprays was characterized in an adult male nasal cast (Aeronose™, courtesy of Aptar/DTF/Univ. of Tours) with chemical quantification. The nasal cast model used was designed from Computed Tomography images (CT scans) of a plastinated head model and anatomically segmented into different regions of interest, including the anterior region, olfactory region, middle and lower turbinates, and rhinopharynx. To ensure accurate regional quantification and prevent cross-regional leakage, the model was segmented using vertical cuts within the 3D-printed blocks and sealed with silicone joints between adjacent sections. The central portion of the model was further divided into three compartments, with the upper third corresponding to the olfactory region. The external nose was manufactured from a flexible silicone material to allow for realistic testing with different nasal devices and administration configurations. The reconstruction, anatomical segmentation, and subsequent modifications of the model were reviewed and validated by a medical specialist [30]. Following development of the physical model, the deposition quantification methodology was optimized through the selection of a suitable marker. Several candidates were evaluated based on adsorption to model materials, solubility, analytical sensitivity, and their ability to maintain physicochemical characteristics comparable to the original formulations, and fluorescein sodium was selected as a surrogate marker. The quantification method was validated for linearity, precision, and stability. Finally, the predictive capability of the nasal cast was validated by comparing in vitro regional deposition results with in vivo scintigraphy deposition data obtained in six healthy volunteers using two different nasal delivery devices, demonstrating good correlation between the model and clinical observations [31]. Each nostril received a single manual actuation of the device into the nasal cast at a fixed horizontal plane angle of 45°, slightly tilted towards the septum by 5°. This administration angle was selected based on previous studies demonstrating enhanced deposition in the olfactory region and turbinates while remaining consistent with the instructions commonly provided in patient information leaflets for nasal spray administration [32,33]. For the initial comparison of the different spray devices using a fluorescein solution, an insertion depth of 15 mm was selected. This depth corresponds to the integrated nostril stop of the Neurospray™ device and represents an optimized administration configuration, as previous studies have shown that increasing insertion depth generally enhances deposition in the upper nasal cavity and olfactory region [34,35]. The 15 mm insertion depth was therefore used to maximize the discriminatory power of the model and assess the performance of the novel devices under best-case administration conditions. To evaluate the final combination products under more conservative conditions, a second set of experiments was performed using an insertion depth of 8 mm, representing a worst-case administration scenario more reflective of suboptimal patient use. This approach allowed for an assessment of the device robustness across a range of clinically relevant administration conditions. No airflow was used during testing. The different regions of interest (anterior region, olfactory region, turbinates, lower turbinates, rhinopharynx) and a filter (corresponding to the potential exposure to the lungs) were rinsed with volumes of water adjusted to ensure their complete coverage. Those samples were then quantified on a spectrophotometer (Light Wave II, Bioserv, Morangis, France) with a calibration curve ranging from 0.1 to 10 µg/mL. All analyses were conducted in triplicate. Results are expressed as the percentage of the administered dose recovered in each region of interest, following normalization to the total recovered dose.

### 2.4. AmyP53 Peptide Characteristics

AmyP53 is an adaptive 12-mer synthetic peptide derived from the respective ganglioside-binding domains of α-Synuclein and Aβ [36,37]. It was specifically designed for optimal solubility in water to avoid any complications during the formulation of our therapeutic solution. It is highly soluble in water (up to 200 mg/mL) [25]. AmyP53 (purity >98%, free of endotoxin and residual solvents) was synthesized by Proteogenix (Schiltigheim, France). The peptide was solubilized in ultrapure water (pH 6.9–7.0; resistivity > 18 MΩ·cm), as it was intended for the clinical trial and further administration in patients. AmyP53 contains a tyrosine residue, which allows for the quantitative detection of the peptide at 280 nm [38] without interfering with fluorescein (λ_max_ = 515 nm without absorption at 280 nm). AmyP53 was also detected in biological extracts by Liquid Chromatography–Mass Spectrometry (LC-MS) as previously described [25] and by Enzyme-Linked Immunosorbent Assay (ELISA) [39].

### 2.5. AmyP53 Activity on Ganglioside Monolayers

Peptide–ganglioside interactions were studied by Langmuir film balance measurements using a fully automated microtensiometer (µTROUGH SX, Kibron Inc., Helsinki, Finland). GM1 ganglioside (#1061 Matreya) was dissolved in chloroform:methanol (1:1, v:v) at 1 mg/mL and spread at the air–water interface to form the monolayer. The subphase consisted of ultrapure water (pH 6.9–7.0; resistivity >18 MΩ·cm), and experiments were performed at 20 °C in triplicate. Peptide injection into the subphase induced a time-dependent increase in surface pressure, reflecting insertion into the monolayer. Data were analyzed using FilmWareX (Kibron Inc., Helsinki, Finland).

### 2.6. Nose-to-Brain Administration of AmyP53 in Rabbits

The in-life phase was performed under protocol ALS231240 at OcciLife (Fontenilles, France). Animal housing and care complied with the recommendations of Directive 2010/63/EU. The OcciLife animal facility holds authorization number E 31 188 001, issued by the French Veterinary Authorities, and its animal care and use program is AAALAC-accredited. Three cohorts of three female rabbits aged 10 weeks at the beginning of the treatment with weights between 2 and 3 kg were used in this study. The animals were acclimatized for 7 days prior to experimentation, with water and food provided ad libitum. Each animal received AmyP53 (100 mM) intranasally with the Neurospray™ device, for a total of 100 μL of AmyP53 per animal (2 pushes of 25 µL were deposited in each nostril). The use of the spray device allows for animal handling without anesthesia. Blood samples were collected after AmyP53 administration according to the experimental groups: 10 min, 20 min, or 24 h after the last pulse. Animals were euthanized (electrical stunning) immediately after blood collection. Brains were collected upon sacrifice and processed for AmyP53 detection by High-Performance Liquid Chromatography (HPLC) (one brain hemisphere) and quantitation by ELISA (the contralateral hemisphere).

### 2.7. Detection and Quantitation of AmyP53 in Rabbit Brain and Whole Blood

For HPLC detection, blood and brain samples were extracted at 4 °C at a ratio of 1 g of brain tissue for 2 mL of phosphate-buffered saline (PBS) (pH 7.4) in the presence of aprotinin (50 µg/mL). An amount of 70 µL of each sample was then transferred into low-binding tubes and treated with 12 µL 8% ascorbic acid in acetonitrile (ACN) 2% for protein extraction, and then with 10 µL of internal standard (irbesartan) solution at 0.5 µg/mL. The samples were treated successively with 10 µL of ZnCl solution, sonicated for 30 s, and finally mixed with 100 µL of ACN and centrifuged at 20,000 *g* at 4 °C for 15 min. An amount of 90 µL of supernatant was then mixed with 90 µL of H_2_O, after which 20 µL of 1% formic acid (FA) was added. After gentle mixing, the samples were centrifuged at 2000 *g* at 4 °C for 5 min. An amount of 10 µL was injected into the chromatography system (AB Sciex API 6500, with an analytical column XBridge BEH C18 4.6 × 150 mm 3.5 µm). The mobile phase was H_2_O + 0.01% NH_4_OH/ACN + 0.5% Formaldehyde. Blood samples (exactly 1.0 mL) were collected into K3-EDTA tubes containing 10 µL of protease inhibitor from the jugular or auricular veins of rabbits.

For AmyP53 quantification, brain hemispheres were first homogenized in phosphate buffer containing EDTA and protease inhibitors, as previously described [25]. Two volumes of methanol were added to one volume of brain homogenate and incubated at 4 °C for 1 h. The extract was centrifuged at 16,000× *g* for 10 min at 4 °C. The supernatants were serially diluted in phosphate buffer for ELISA quantitation of AmyP53. For LC-MS, the brain homogenates were treated with ACN and trifluoracetic acid before injection into the column. 13-C/15-N AmyP53 (purity >90%, purchased from Abyntek, Bizkaia, Spain) was used as an internal standard.

### 2.8. Bioanalytical Method Validation

The bioanalytical methods used for AmyP53 quantification in this study were validated according to standard criteria (specificity, linearity, precision, limits of detection/quantification), as summarized below and detailed in the Supplementary Materials.

#### 2.8.1. Quantification of AmyP53 in Biological Matrices

Assay validation was performed in rabbit brain and rabbit whole-blood matrices. Calibration curves were established using a weighted (1/X^2^) linear regression model (Response = Slope × Concentration + Intercept) over the range of 100–20,000 ng/mL. Regression parameters, linearity, and limits of quantification for each matrix are summarized in Supplementary Table S1. Assay precision, expressed as the coefficient of variation (CV%) calculated from duplicate measurements at representative concentration levels across the calibration range, is presented in Supplementary Table S2. Assay specificity was confirmed by the absence of a detectable peptide signal in blank matrix samples (Table S1). In the whole-blood matrix, no chromatographic peak could be detected below the LLOQ (see Figure 5D); in the brain matrix, an indicative detection threshold below the validated lower limit of quantification (LLOQ) was estimated by peak extrapolation (Table S1).

#### 2.8.2. Reference Standard Characterization by HPLC-UV

The AmyP53 standard solution (1 mM in water) was independently characterized by HPLC-UV. Serial dilutions were injected in triplicate (5–100 pmol), and the peak area was plotted against the injected amount, yielding a linear calibration curve (Figure S1A). The regression parameters, limit of detection, recovery, and precision extracted from this calibration curve are summarized in Supplementary Table S3. The stability of the AmyP53 formulation upon prolonged storage was previously established by HPLC-UV analysis (Figure S1B–E) [25].

#### 2.8.3. ELISA Assay

The validation of the AmyP53 ELISA has been previously described [39], and the corresponding calibration curve is shown in Figure S2.

### 2.9. Statistical Analysis

Statistical comparisons between groups were performed using one-way analysis of variance (ANOVA), followed by unpaired t-tests where appropriate, with Bonferroni correction applied to account for multiple comparisons. All statistical analyses were performed using JMP 19.0.4 (JMP Statistical Discovery LLC, Cary, NC, USA). Probability values of <0.05 were considered statistically significant. Group sizes and *p*-values are reported in each figure legend.

## 3. Results

### 3.1. Comparative Analysis of Several Sprays for Nose-to-Brain Delivery

To identify the most suitable sprays for AmyP53 delivery to the brain, six devices were compared: four commercial non-targeting devices (commercial devices 1–4) and two specifically designed for nose-to-brain delivery based on different technologies (the Neurospray™ and Neurospray™ PF).

**Figure 1:**
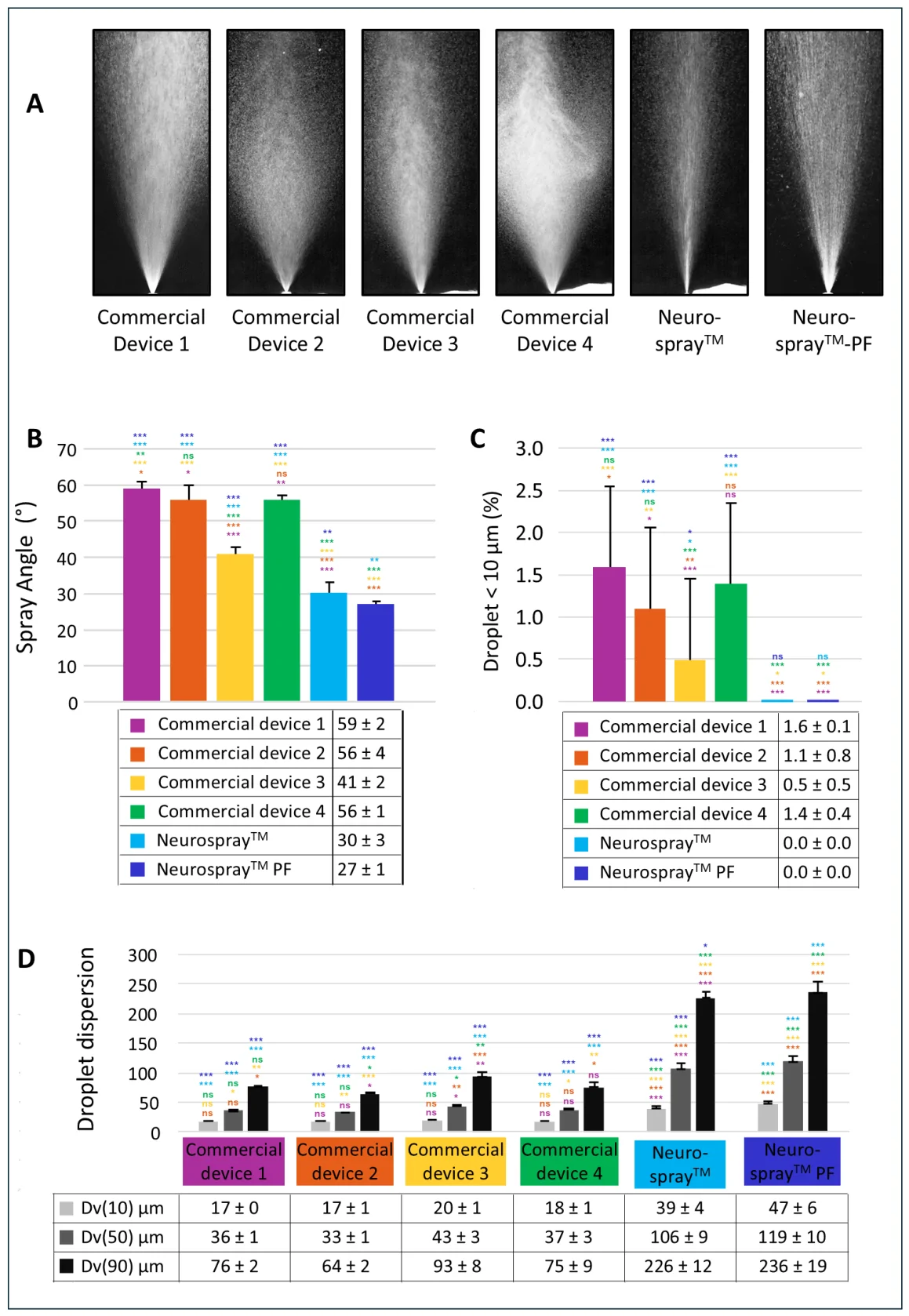
A comparison of the plume angles of six nasal spray devices. Six nasal devices were investigated for their spray properties. (**A**) Representative pictures of the sprayed liquid through the different devices. (**B**) Quantification of the spray angle. (**C**) Quantification of the proportion of small droplets (<10 µm). (**D**) Repartition of the droplet size distribution, the average of 10%, 50% (volume median), and 90% of the cumulative volume undersize (Dv10, Dv50, and Dv90, respectively) during the fully developed phase of the spray. Results are represented as mean ± SD (*n* = 10). ANOVA multiple-comparison tests were performed (* *p*-value < 0.05; ** *p*-value < 0.01; *** *p*-value < 0.001. ns: no significant difference). The star colors indicate the data used for histogram comparison: violet is compared to commercial device 1, orange to commercial device 2, yellow to commercial device 3, green to commercial device 4, light blue to the Neurospray^TM^ and dark blue to the Neurospray^TM^-PF.

The first performance assessment consisted of measuring two different parameters of the spray: the size of the droplets and the angle of the spray (Figure 1A,B). Droplet size is a critical determinant of intranasal and nose-to-brain delivery because larger droplets could deposit in the anterior nasal cavity due to inertial impaction, whereas droplets in the respirable range (<10 µm) may bypass the nasal cavity and deposit in the lungs, which is undesirable for safety [34,40,41,42]. For all devices, the percentages of droplets <10 µm (referred to as % < 10 µm) are minimal (Figure 1C), meaning that the risk of lung exposure is very low. Three of the marketed actuators (commercial devices 1, 2 and 4) demonstrated equivalent Dv50s (volume-based diameters below which 50% of the droplets fall) (Student’s t-test, *p* > 0.05) and spray angles, with a slight statistical difference between devices 1 and 2 (*p* = 0.02). These devices had smaller droplet size distributions and larger spray angles compared to the third automated actuator (commercial device 3). Lastly, two Neurospray™ devices (referred to as the Neurospray™ and Neurospray™-PF) produced significantly larger droplets than the four marketed actuators tested. As shown in Figure 1D, the Dv90s (volume-based diameters below which 90% of the droplets fall) exceed 200 µm for both Neurospray™ devices, whereas it is approximately 100 µm or lower for all other commercial devices tested. Additionally, the spray angles of the two Neurospray™ delivery systems were significantly narrower than that of any other actuator tested (Figure 1A,B). In summary, both Neurospray™ devices exhibit larger droplets and narrower spray angles, the latter being a key feature for efficient nose-to-brain targeted drug delivery.

### 3.2. Evaluation of the Spray Devices with the Nasal Cast

**Figure 2:**
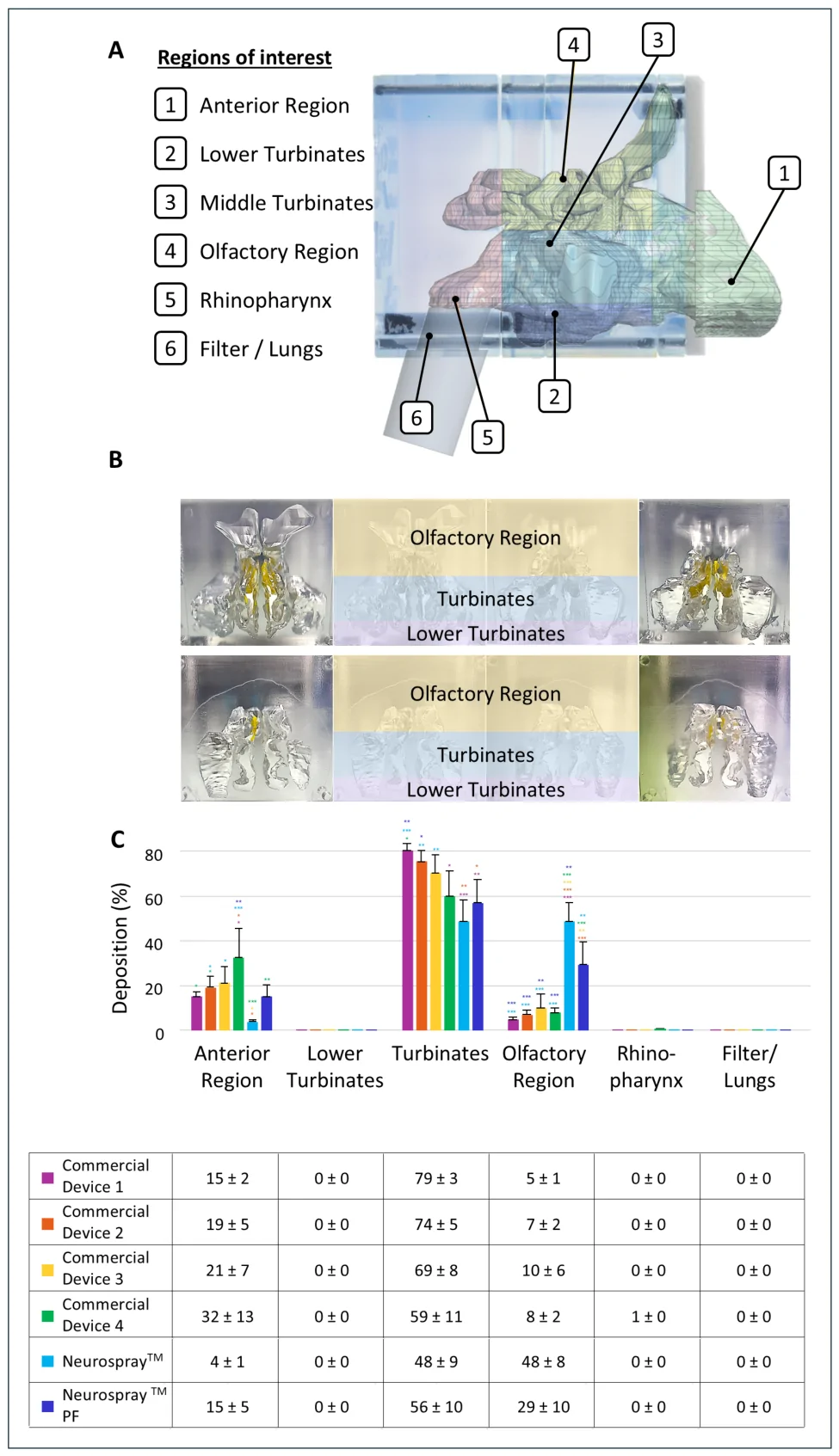
Solution deposits for the different devices using the nasal cast system. (**A**) Schematization of the human nasal cast with the different represented regions—anterior region, olfactory region, turbinates, lower turbinates, rhinopharynx—and a filter corresponding to the potential exposure to the lungs. (**B**) Images of the nasal cast showing front and back views of the two central blocks encompassing the olfactory region and the middle and lower turbinates following Neurospray™ actuation, with fluorescein-covered regions highlighted in yellow. (**C**) An aqueous 75 mg/mL fluorescein sodium solution was sprayed at a single insertion of 15 mm. The different parts of the nasal cast were rinsed with volumes of water adjusted to ensure complete coverage of the different regions of interest. Those samples were then quantified by spectrophotometry. Results of the deposition of the spray in the different nasal cast regions according to the 6 nasal sprays used at an insertion depth of 15 mm are shown. Results are represented as mean ± SD (*n* = 3). ANOVA multiple-comparison tests were performed. * *p*-value < 0.05; ** *p*-value < 0.01; *** *p*-value < 0.001. The star colors indicate the data used for histogram comparison: violet is compared to commercial device 1, orange to commercial device 2, yellow to commercial device 3, green to commercial device 4, light blue to the Neurospray^TM^ and dark blue to the Neurospray^TM^-PF.

After the determination of the spray performances of the six devices, we investigated their ability to target the olfactory region for optimal nose-to-brain delivery. For these experiments, we used the nasal cast apparatus, which comprises different regions of interest (anterior region, olfactory region, turbinates, lower turbinates, rhinopharynx) and a filter corresponding to the potential exposure to the lungs (Figure 2A,B).

The human nasal cast allows for the precise evaluation of the amount of product delivered into each target region. Figure 2C present the regional deposition results for the four commercial non-targeting devices and the two Neurospray™ delivery systems, using an aqueous 75 mg/mL fluorescein sodium solution at a single insertion of 15 mm. The Neurospray™ systems showed significantly higher deposition in the olfactory region compared to commercial devices 1–4 (ANOVA, *p* < 0.05), with minimal losses in the anterior region.

These findings indicate that lower micronization of the liquid (resulting in a larger droplet size and smaller spray angle) leads to reduced losses in the anterior region and more effective targeting of the olfactory region [34]. Thus, the two Neurospray™ devices, which produce less micronized sprays with smaller plume angles, target the olfactory region with higher efficiency than the commercial actuators herein tested.

## 4. Evaluation of Neurospray™ Devices with AmyP53 Formulation

**Figure 3:**
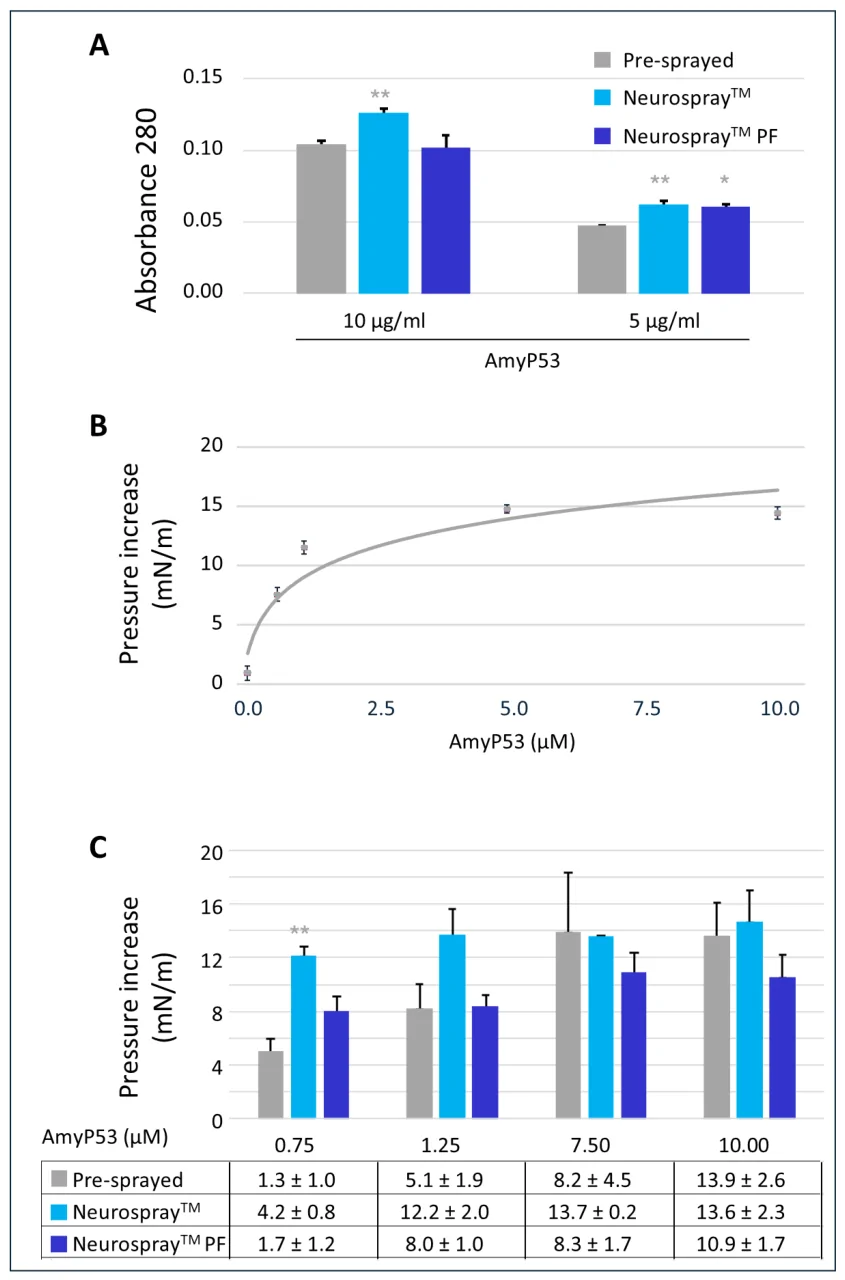
Evaluation of the integrity of the AmyP53 molecule after spraying. An AmyP53 solution (100 µM) was placed into the two Neurospray™ devices. After priming of the devices, the sprayed solutions were collected to be tested. (**A**) Absorbance at 280 nm of the 10 µM and 5 µM diluted solutions. (**B**,**C**) Ganglioside monolayers prepared at the air–water interface at an initial surface pressure value of 17.5 mN.m^−1^. After stabilization, AmyP53 was injected into the aqueous subphase at the indicated final concentration. The data are expressed as the average maximal surface pressure increase induced by the peptide at each concentration. (**B**) Scale of the maximal surface pressure obtained after injection of AmyP53 at different concentrations under a GM1 monolayer (n = 3). (**C**) Biological activity of AmyP53 delivered by the Neurosprays™. AmyP53 pre-spray (gray histograms), AmyP53 delivered by the Neurospray™ (light-blue histograms) or AmyP53 delivered by the Neurospray™-PF (dark-blue histograms) were injected underneath a monolayer of ganglioside GM1 prepared at the air–water interface. The data show the maximal surface pressure increase induced by the peptide. No statistical difference could be observed between standard and sprayed AmyP53 (*n* = 3), demonstrating a 100% conservation of biological activity of AmyP53 delivered by the spray device. Results are represented as average ± SD (*n* ≥ 3). ANOVA multiple-comparison tests were performed. * *p*-value <0.05; ** *p*-value < 0.01. The star colors indicate the data used for histogram comparison; gray is compared to pre-sprayed solution.

From all our previous tests, we determined that the two Neurospray™ devices were the best candidates for the nose-to-brain delivery. Thus, these Neurospray™ delivery systems were tested with calibrated aqueous solutions of AmyP53. For each concentration, the percentage of AmyP53 actually sprayed by the device was measured by the absorbance at 280 nm and is plotted in Figure 3A. The data showed that the amount of AmyP53 delivered by both Neurospray™ devices was consistently slightly above 100% of the nominal dose at all tested dose levels. This result suggests that AmyP53 was efficiently emitted from these devices, with no evidence of major retention in the reservoir under our experimental conditions. The slight excess over 100% may reflect interfacial enrichment of the peptide in aerosol droplets, as reported for surface-active compounds and, in some cases, for proteins during aerosolization [43,44].

In a second series of experiments, we evaluated the biological activity of AmyP53 delivered by both Neurospray™ devices. In these experiments, the sprayed peptide was incubated underneath a monolayer of ganglioside GM1, and the interaction was followed in real time by surface pressure measurements according to our standard protocol [45]. A calibration curve assessing the ganglioside-binding properties of AmyP53 over the range of concentrations tested was used as a reference (Figure 3B). The results obtained with AmyP53 delivered by the Neurospray™ systems are shown in Figure 3C. At all concentrations tested, the device was able to deliver the totality of biologically active AmyP53 without any statistical decrease. Interestingly, the tendency of the devices to increase the amount of available AmyP53 (Figure 3A) was confirmed by surface pressure measurements (Figure 3C). Overall, these data demonstrate that AmyP53 remains fully stable and retains its biological activity after its delivery by both Neurospray™ drug delivery systems.

### 4.1. The Neurospray™ Devices with the AmyP53 Formulation Result in High Deposition in the Olfactory Region

**Figure 4:**
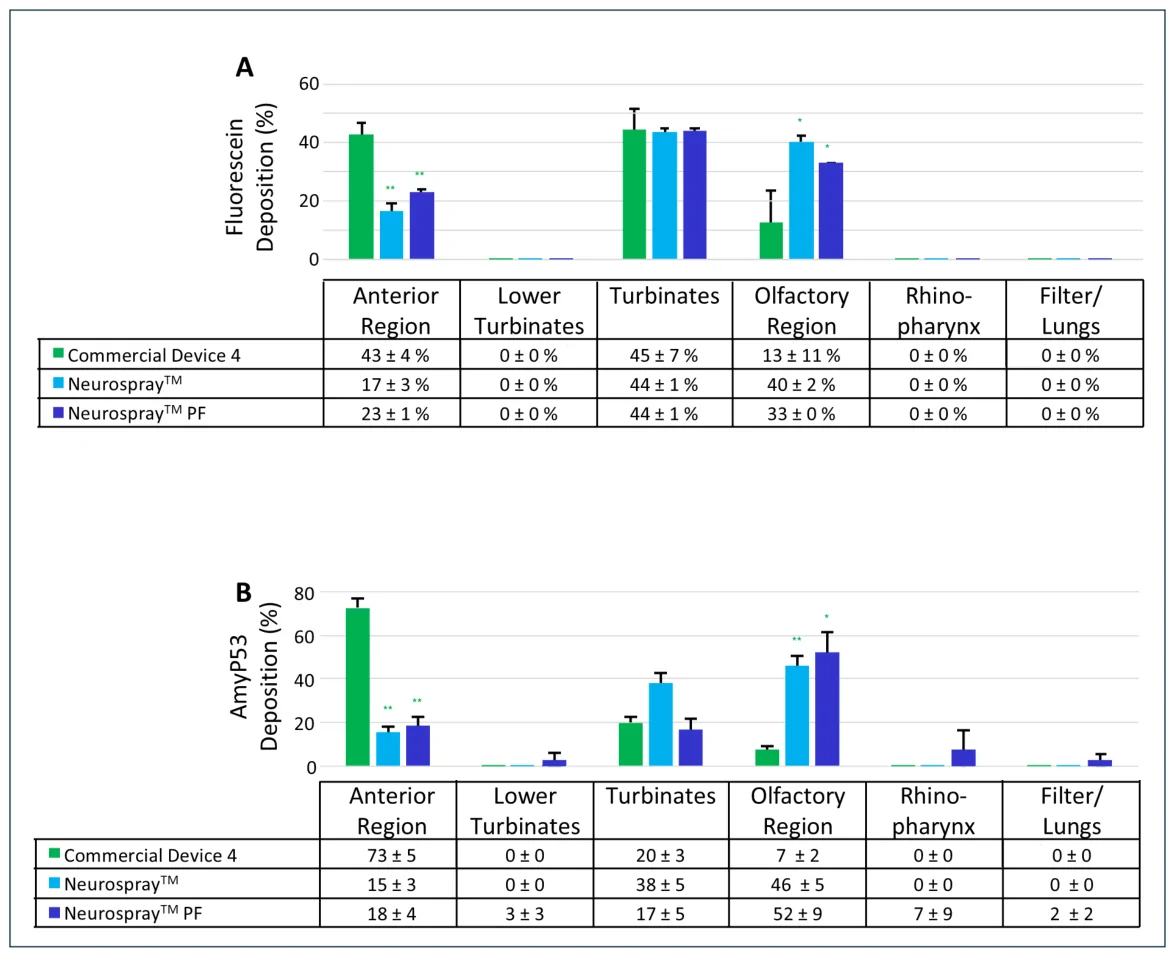
AmyP53 dosages in samples recovered from the nasal cast. An aqueous solution of AmyP53 at 100 mg/mL and 0.7 mg/mL fluorescein sodium solution was sprayed at a single insertion depth of 8 mm using commercial device 4, the Neurospray™ or the Neurospray™-PF. The different parts of the nasal cast were rinsed with volumes of water adjusted to ensure complete coverage of the different regions of interest. Samples were collected for quantification. (**A**) Samples were then quantified by spectrophotometry thanks to the fluorescein signal. (**B**) AmyP53 concentrations were quantified using absorbance at 230 nm. Results are represented as mean ± SD (*n* ≥ 3). ANOVA multiple-comparison tests were performed. * *p*-value <0.05; ** *p*-value < 0.01. The star colors indicate the data used for histogram comparison; green is compared to commercial device 4.

Finally, to ensure that the AmyP53 solution could reach the zones of interest in the brain with both Neurospray™ products, as previously demonstrated with fluorescein used as the control (Figure 2), we sprayed the AmyP53 formulation into the nasal cast apparatus. In this experiment, AmyP53 was tested in the same experimental conditions with commercial device 4 (Figure 4). We quantified on one side the fluorescein (Figure 4A), then the AmyP53 (Figure 4B). Both measurements gave similar results, indicating that the behavior of AmyP53 was identical to that of fluorescein in the nasal cast system. Most importantly, none of the tested sprays exposed the peptide to the lungs, confirming the results obtained with fluorescein alone (compare Figure 2 to Figure 4). Moreover, there was no loss in the rhinopharynx or the lower turbinates, indicating that AmyP53 was chiefly vectorized towards neuronal-exposed regions. The data confirmed the superiority of the Neurospray™ devices over the previous commercial device, as the AmyP53 recovered from the nose region was minimal with the Neurospray™ systems compared with commercial device 4. The amounts of AmyP53 recovered from the olfactory region and turbinates indicate that the nose-to-brain delivery of AmyP53 by the Neurospray™ devices was far more efficient than with commercial device 4 (*p* < 0.01). Specifically, the Neurospray™ and Neurospray™ PF delivered the AmyP53 solution at 84 ± 3% and 77 ± 1%, respectively, measured by fluorescein (Figure 4A) and at 84 ± 3% and 69 ± 14% measured by AmyP53 absorbance (Figure 4B) to the olfactory region and the turbinates. In contrast, these percentages were significantly lower with commercial device 4 (58 ± 18% with fluorescein measurement and 27 ± 5% with AmyP53 measurement).

### 4.2. Preclinical Data in Rabbits Suggest Direct Access to the Brain for the AmyP53 Formulation Combined with a Neurospray™ Device

To estimate the amount of AmyP53 reaching the brain following a single nose-to-brain administration with the Neurospray™, we tested the standard Neurospray™ in rabbits. The choice of this animal model was motivated by (i) the anatomical characteristics of the nasal cavity [1,46], and (ii) the ganglioside composition, which appears closer to that of humans than those of other animal models [26]. As a comparison, the olfactory area in rats represents 50% of the nasal cavity, whereas it is only 10% in rabbits, closer to the human morphology, where it represents only 3–5%. In addition, for intranasal drug delivery, preclinical studies in animals larger than rodents are recommended [47].

**Figure 5:**
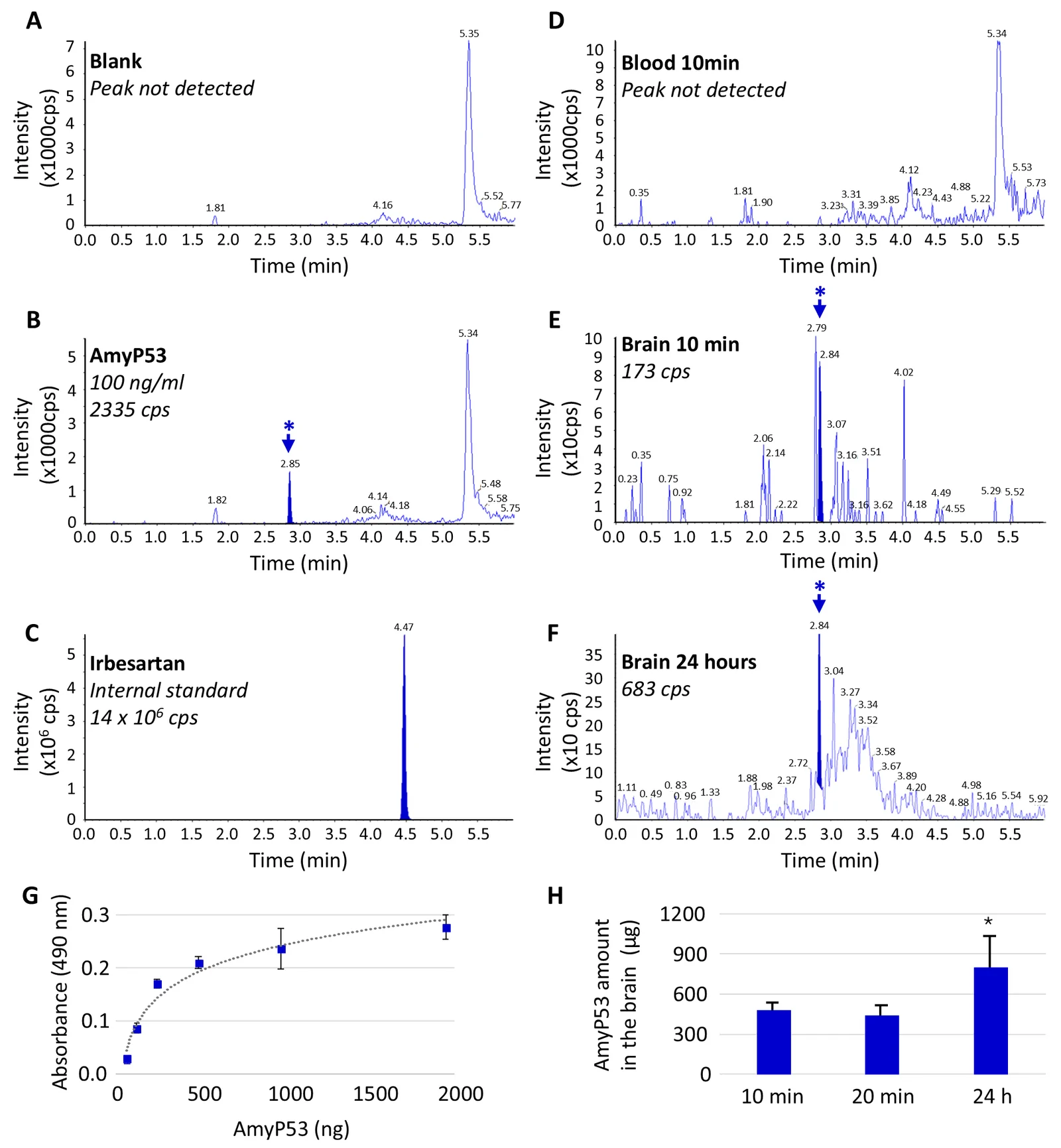
Detection of AmyP53 in brain extracts of rabbits treated with AmyP53 administered intranasally. (**A**–**F**,**H**) Sample analysis using HPLC analysis. When applicable, the intensity of the AmyP53 and Irbesartan peaks is indicated in each chromatogram. Samples are rabbit brain extracts from animals subjected to intranasal administration of AmyP53 (100 µL of 100 mM) or the control (no injection of Irbesartan). (**A**) Control rabbit brain extract (no AmyP53 treatment). (**B**) Rabbit brain extract spiked with AmyP53 with a typical and specific peak at 2.85 min (asterisk). (**C**) Rabbit brain extract spiked with internal standard (Irbesartan), with a typical and specific peak at 4.47 min. (**D**) Lack of detection of AmyP53 in rabbit whole-blood extract 10 min after intranasal administration. (**E**) Detection of AmyP53 in rabbit brain extract 10 min after intranasal administration (the asterisk shows the typical AmyP53 peak). (**F**) Detection of AmyP53 in rabbit brain extract 24 h after intranasal administration (the asterisk shows the typical AmyP53 peak). (**G**) Dose-range detection of standard AmyP53 in the rabbit brain matrix by ELISA. (**H**) Quantitative detection of AmyP53 by ELISA in rabbit brain extracts at the indicated time after intranasal administration of the peptide. Data are expressed as mean ± SD (*n* = 3). ANOVA multiple-comparison tests were performed. * *p*-value < 0.05.

In our experiments, 100 µL of 100 mM AmyP53 was sprayed into the noses of the rabbits (50 µL per nostril). Under these conditions, the peptide was not detected in the blood 10 min after the administration, suggesting that the device delivered the drug efficiently to the brain, with minimal/no systemic exposure (Figure 5). The animals were sacrificed at 10 min, 20 min and 24 h post-treatment, and their full brains were processed for AmyP53 detection and quantification. First, we used HPLC to specifically detect AmyP53 (Figure 5A,B), using Irbesartan as an internal standard (Figure 5C). Under our conditions, the peptide was detected as a single peak at 2.85 min in standard samples (Figure 5B). This typical AmyP53 peak was not detected in blood extracts (Figure 5D), which indicates no or very limited systemic exposure. In contrast, AmyP53 was reproducibly detected in the chromatograms corresponding to brain extracts at 10 min (Figure 5E) and 24 h (Figure 5F) following nose-to-brain administration. The low volume injected into the column (10 µL) did not allow for a precise quantitation of AmyP53 because it was under the lower limit of quantification (100 ng/mL). Thus, we decided to quantify the amount of AmyP53 recovered from brain extracts by ELISA [25]. A representative calibration curve is shown in Figure 5G. This method allowed us to detect any amount of AmyP53 in rabbit brain extracts above 60 ng. The amounts of AmyP53 detected in the brains of rabbits treated with the Neurospray™ were determined at 10 min, 20 min, and 24 h (Figure 5H). These data confirmed the results obtained by the HPLC method and allowed us to accurately determine the amounts of AmyP53 targeting the brain. Given the volume of brain extract and the ELISA conditions (described in the Section 2.7), we could extrapolate the total amounts of AmyP53 in the brain to 480 ± 54 µg, 440 ± 79 µg, and 800 ± 232 µg for time-points of 10 min, 20 min, and 24 h, respectively. Overall, these amounts represented 3.8%, 3.5%, and 6.4%, respectively, of the dose initially injected (detailed calculations in the Supplementary Materials). The highest amount of peptides was detected after 24 h. While this later signal could reflect successive waves of AmyP53 delivery according to the two penetration mechanisms discussed below, alternative explanations cannot be excluded, including tissue retention of the peptide or a slow-release phenomenon from the nasal mucosa or brain tissue. Distinguishing between these possibilities will require dedicated kinetic and mechanistic studies, and the successive-wave hypothesis should therefore be considered speculative at this stage. Nevertheless, the detection of AmyP53 in the whole-brain homogenate means that it reaches its intended target (the brain), which is the key requirement for achieving therapeutic benefit in patients with Alzheimer’s or Parkinson’s disease.

## 5. Discussion

**Figure 6:**
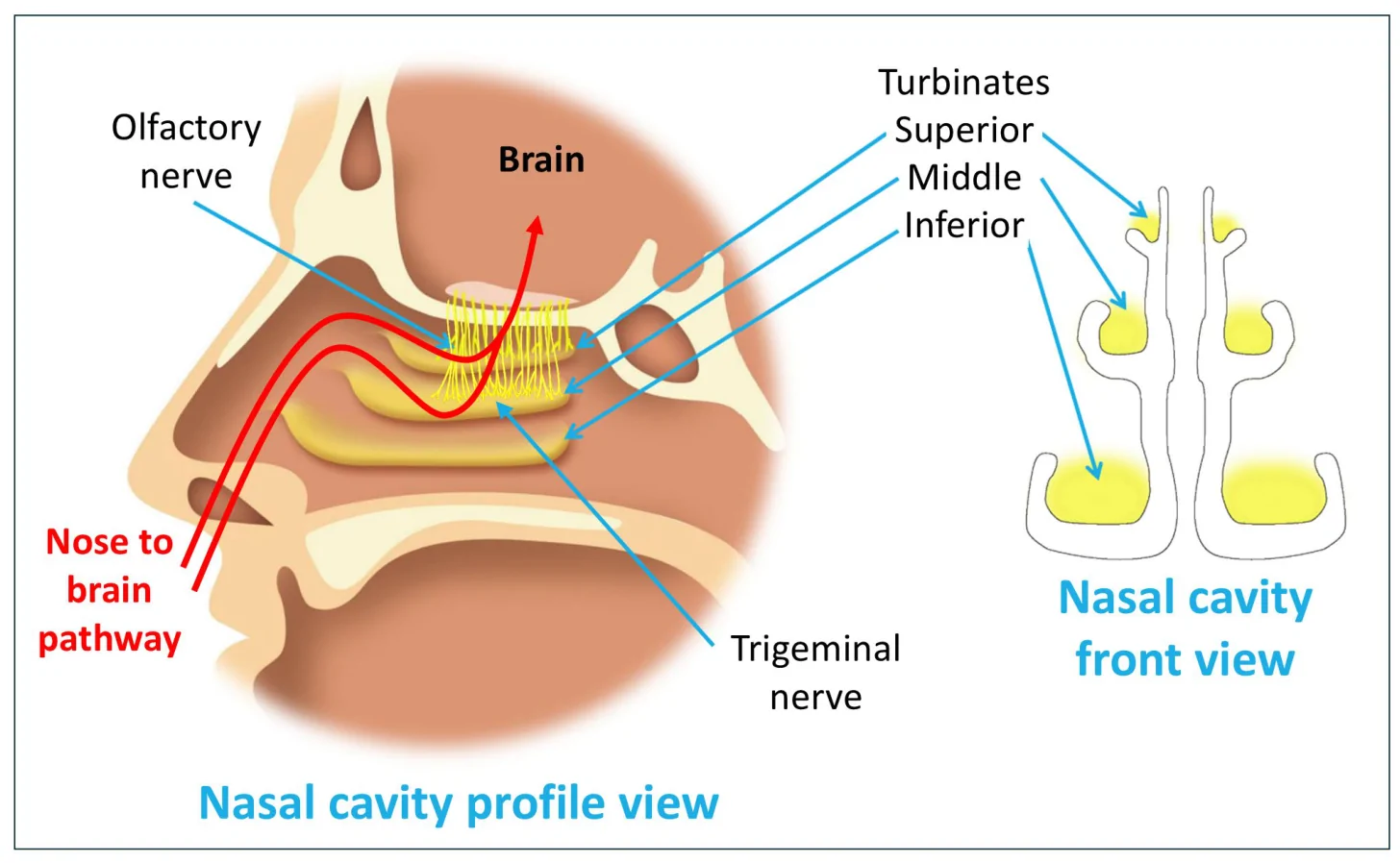
A schematic representation of the different regions of the nasal cavity. The schematic locates the olfactory nerve as well as the turbinates, the targets for nose-to-brain delivery (reproduced and modified from [53]).

The choice of delivery device is a major clinical challenge that needs to be carefully addressed when developing a new therapeutic compound. For neurodegenerative conditions like Alzheimer and Parkinson’s diseases, the nose-to-brain pathway offers particular promise [1,48,49,50]. This noninvasive route utilizes the unique anatomical connection between the nasal cavity and the brain through the olfactory and trigeminal nerve pathways, bypassing the blood–brain barrier [2,48,51,52]. The olfactory nerve is located in a region at the top of the nasal cavity (olfactory region), and its neurons are directly exposed to the nasal cavity in an area with limited mucociliary clearance (Figure 6). In contrast, the trigeminal nerve is spread across the turbinates, which have a higher surface area and exposure to the bloodstream, allowing for fast systemic absorption.

Among the critical parameters, it is of particular importance to ensure that the olfactory zone and upper turbinates are efficiently targeted by therapeutic drugs. In the present study, we evaluated novel devices for optimal drug delivery to the brain: the Neurospray™ and Neurospray™ PF. Then, we analyzed the performances of these devices for the therapeutic peptide AmyP53, which targets a neurotoxicity mechanism common to Alzheimer’s and Parkinson’s diseases. The first part of this study consisted of determining the characteristics of the new devices and comparing them with commercial devices. In particular, we focused on three critical parameters: (i) droplet size, (ii) plume angle, and (iii) regional deposition. To ensure successful deposition within the nasal cavities, a certain degree of droplet micronization is required to promote distribution and coverage within the target regions while enabling impaction without causing discomfort. However, these droplets should not be excessively fine (<10 µm), as finer droplets may penetrate deeper into the respiratory tract and reach the lungs [42]. Our results showed that the six devices analyzed in this study fulfilled this criteria, with Dv50s within 36–119 µm and % < 10 µm lower than 2%. Next, we measured the plume angle of each device, which should ideally be the narrowest possible to deliver the optimal dose to the olfactory region. Using this parameter, we could determine that the most performant devices for targeted delivery were the two Neurospray™ devices with a plume angle of 30° (Figure 1). The regional deposition was then tested in our nasal cast system (Figure 2). We found that the Neurospray™ systems were far more efficient than the four other conventional commercial devices at targeting the olfactory region. Thus, the Neurospray™ drug delivery systems were clearly two good candidates for the intranasal administration of the therapeutic AmyP53 peptide in clinical trials.

In a second step, we verified the performances of the devices with an AmyP53 peptide solution (Figure 4). Regardless of the concentration analyzed in a 0.2–10 µM range, the totality of the peptide was recovered in the vaporized droplets. In addition, with each Neurospray™, the aerosolized peptide retained its biological activity, i.e., its ability to physically interact with its therapeutic target, i.e., brain cell gangliosides. This property was verified using a specific peptide–ganglioside-binding assay developed by our team [36,54]. This is based on the reconstitution of a monolayer of gangliosides at the water–air interface and quantifying the binding of the AmyP53 peptide by surface pressure measurements. Based on these results, we then prepared a mixed solution containing fluorescein and AmyP53 to study the behavior of these two compounds in the nasal cast (Figure 3). The results obtained with AmyP53 were similar to those of fluorescein, with maximum administration to the regions exposed to brain neurons (especially the olfactory region) and very little or none (less than 5%) to the respiratory system.

It is important to mention that AmyP53 is an adaptive peptide derived from intrinsically disordered regions of α-Synuclein (associated with Parkinson’s disease) and the Aβ protein (associated with Alzheimer’s disease) [36]. It remains unstructured in aqueous solution and only adopts a defined conformation upon interaction with its molecular target, gangliosides, in line with the “target the target, not the arrow” concept previously proposed for this class of therapeutic peptides [37]. As a result, conventional secondary-structure techniques are not applicable to assessing peptide integrity in the absence of target engagement. The ganglioside-binding assay used in this study, based on real-time Langmuir monolayer measurements, therefore represents the most relevant functional readout for evaluating the preservation of AmyP53 activity after aerosolization, as it directly probes the target-dependent interaction that underlies the peptide’s therapeutic mechanism [20]. While functional assays such as calcium flux measurements have previously been used to demonstrate that AmyP53 inhibits amyloid oligomer-induced neurotoxicity in its non-aerosolized form [21,22,25,39,55], these assays were not performed on the aerosolized peptide in the present study. Confirming that this downstream functional activity is fully preserved after aerosolization therefore remains an important objective for future work, complementary to the ganglioside-binding data reported here.

With all these device validation criteria established, we then used the device under “real-life” conditions in vivo. For the animal model, we chose the rabbit, whose nasal anatomy is closer to that of humans and is therefore recommended for preclinical regulatory studies of molecules administered via the nose-to-brain route. For all these reasons, rabbits have been chosen as the preferred preclinical animal model for intranasal insulin administration [56], confirming our choice of animal model. For the in vivo part of our study, rabbit also has the advantage of expressing a repertory of gangliosides that closely mirrors human composition, with low contents of the sialic acid Neu5Gc, unlike other species [26]. Thus, rabbit is a reliable preclinical model for testing the pharmacological properties of AmyP53, a ganglioside-targeting therapeutic peptide [21].

In this study, using the nose-to-brain Neurospray™ device in rabbits, we were able to demonstrate the direct access to the brain of AmyP53 (Figure 5). The peptide was reproducibly detected in the brain at 10 min, 20 min and 24 h. While the present study design did not include perfusion controls, these findings are consistent with our previous pharmacokinetic study in rats [25], in which we compared AmyP53 levels in intact versus perfused brain tissue to exclude blood-derived contamination. That study confirmed the genuine parenchymal exposure of AmyP53 following intranasal administration, independent of residual peptide in the cerebral vasculature, supporting direct and/or indirect nose-to-brain transport, as previously described [25]. Taken together with the present rabbit data, these results support brain exposure to AmyP53 after intranasal administration; however, we acknowledge that additional matrix-specific controls (e.g., perfused brain tissue, olfactory bulb quantification) within the rabbit model itself would further strengthen the direct evidence of nose-to-brain transport in this species.

Interestingly, the amount of AmyP53 detected in the brain was greater after 24 h than after 10 and 20 min, suggesting that the peptide may reach the brain by successive waves involving distinct mechanisms. It should be emphasized that the following mechanisms are proposed based on previous studies conducted with other intranasally administered molecules and were not directly investigated in the present study; their involvement in AmyP53 brain delivery therefore remains hypothetical at this stage. Indeed, there are three different pathways through which the peptide may pass the olfactory epithelium and get access to brain tissue: paracellularly between epithelial cells, transcellularly through epithelial cells, and intracellularly through nerve-mediated pathways. (1) In the paracellular pathway mechanism, drugs traverse the spaces between the epithelial cells of the nasal mucosa [57]. This pathway primarily allows for the movement of small molecules and hydrophilic drugs through tight junctions between supporting cells in the olfactory epithelium. After passing through these intercellular spaces, drugs can reach the lamina propria and then access the perineural space, ultimately entering the cerebrospinal fluid (CSF) and the brain. The paracellular route is generally faster than the intracellular (axonal) route, with drug absorption into the brain and CSF occurring within minutes after nasal administration. (2) The transcellular pathway describes the vectorial transport of drugs through the nasal epithelium. It is generally considered the most suitable for lipophilic and low-molecular-weight molecules [11]. (3) In the intracellular mechanism, drugs are internalized by olfactory sensory neurons via endocytosis or pinocytosis [9]. Once inside the neuron, drugs are transported along the axon (axonal transport) to the olfactory bulb in the brain, where they are released via exocytosis. This process is then repeated across synapses, potentially allowing drugs to reach deeper brain regions. The intracellular pathway is typically slower, with axonal transport rates ranging from hours to a day, depending on the drug and neuronal transport dynamics. This mechanism involves two major cranial nerves: the olfactory nerve and the trigeminal nerve [58]. After crossing the olfactory mucosa, drugs are absorbed by olfactory neurons and transported directly to the olfactory bulb, bypassing the blood–brain barrier. Moreover, the trigeminal nerve innervates both the respiratory and olfactory regions of the nasal cavity. Drugs can be transported along its branches to the brainstem and other brain regions. Both nerve-mediated pathways involve perineural and perivascular transport, where drugs move along the nerve sheaths and associated vascular spaces into the central nervous system. Correspondingly, direct nose-to-brain transport via olfactory and/or trigeminal pathways, bypassing the blood–brain barrier, has been previously established for several other therapeutic peptides and proteins, including IGF-1, interferon-beta, and various neurotrophic factors [59,60,61,62]. Notably, the challenge of distinguishing direct olfactory transport from systemic, blood-mediated brain exposure following intranasal administration has also been reported for other peptide candidates [63], underscoring that this represents a broader methodological consideration in the field of intranasal peptide delivery. As AmyP53 shares comparable physicochemical characteristics with these previously studied molecules, in particular its peptidic nature, molecular size, and route of administration, the same olfactory and trigeminal pathways represent plausible candidate mechanisms contributing to its brain delivery.

Overall, our data suggest that AmyP53 is potentially delivered to the brain via a combination of these three mechanisms, thus accounting for the successive waves of the peptide detected in rabbit brains. This hypothesis is also supported by previous pharmacokinetics data obtained for cannulated rats [25]. Yet, it is important to note that significant amounts of AmyP53 were detected in the blood of cannulated rats after intranasal administration [25], whereas this was not the case in rabbits treated with the Neurospray™ device, in which AmyP53 was detected essentially in brain tissue. We attribute this difference primarily to the mode of intranasal administration rather than to a species-specific effect. In the rat study, the small size and anatomical constraints of the nasal cavity precluded the use of a spray device, and AmyP53 was therefore administered via surgically implanted intranasal cannulas; this method of administration likely resulted in partial nasal drip, swallowing, and/or direct mucosal absorption into the systemic circulation, accounting for the detectable blood levels observed. In contrast, the Neurospray™ device used in the present rabbit study was specifically engineered to produce a narrow plume angle (~30°) and an optimized droplet size distribution, achieving high-precision deposition in the olfactory region (up to 84%), as determined in a validated human nasal cast. This targeted deposition likely minimized systemic absorption via swallowing or non-target mucosal routes, consistent with the absence of detectable AmyP53 in rabbit blood despite clear brain exposure. While these observations are consistent with the intended nose-to-brain delivery strategy of the Neurospray™ device, we acknowledge that direct cross-species comparisons should be interpreted with caution, given the differences in the administration methods, dosing normalization, and sampling protocols between the rat and rabbit studies. Further studies using comparable administration routes across species would be needed to formally confirm this interpretation.

In this respect, the present study has several limitations that should be acknowledged. First, the biodistribution analysis was performed on a small cohort of three animals per time point, in accordance with the 3Rs principle, to minimize animal use in this exploratory study; larger cohorts will be required to confirm these findings with greater statistical confidence. Second, as we performed whole-brain homogenate analysis rather than region-specific brain distribution analysis, it is not possible to confirm that the olfactory region is the only region, or the predominant region, targeted by nose-to-brain delivery using the Neurospray™. Third, this study did not include a direct in vivo comparison between the Neurospray™ device and a conventional nasal delivery system or an alternative route of administration; such a comparison will be necessary in future studies to precisely quantify the improvement in brain targeting provided by the Neurospray™ over existing approaches. Despite these limitations, the present study provides compelling proof-of-concept evidence that the Neurospray™ device enables the efficient, noninvasive, and reproducible brain delivery of AmyP53 following intranasal administration, establishing a solid foundation for its advancement into the planned Phase 1 clinical trial. Indeed, the previous nose-to-brain delivery system allowed less than 10°% of the therapeutic solution to reach the brain, whereas we reached >80% using the Neurospray^TM^ for AmyP53 delivery to the brain.

## 6. Conclusions

Taken together, these results indicate that the Neurospray™ device achieves highly targeted olfactory region deposition, consistent with the efficient nose-to-brain delivery of AmyP53 in the rabbit model, while minimizing systemic exposure. Although direct cross-species comparisons with our earlier rat pharmacokinetic study should be interpreted with caution given differences in the administration methods, these findings reinforce the rationale for a nose-to-brain delivery strategy for AmyP53. In addition, the long-term stability of the AmyP53 aqueous formulation used in the Neurospray™ device has already been established in a dedicated study, demonstrating stability for up to 18 months at 45 °C [25]. Together with the data reported here, this confirms that the AmyP53 nasal spray formulation is fully qualified for advancement toward clinical development. In conclusion, our study supports the use of both the Neurospray™ and Neurospray™ PF as nasal delivery devices for AmyP53, intended to prevent and/or treat Alzheimer’s and Parkinson’s diseases in future clinical trials.

## 7. Patents

AmyP53 is patented (Patent # EP14305353.6 accepted in the EU, Canada, the US, China and Japan).

## Data Availability

The original contributions presented in this study are included in the article/Supplementary Material. Further inquiries can be directed to the corresponding author.

## Supplementary Materials

The following supporting information can be downloaded at: https://www.mdpi.com/article/10.3390/pharmaceutics18080987/s1, Calculation of the percentage of AmyP53 detected in rabbit brain following intranasal administration. Figure S1: Stability of AmyP53 formulation, as assessed by HPLC-UV [25]. Figure S2: Dosing of AmyP53 by ELISA [39]. Table S1: Bioanalytical method validation parameters for AmyP53 quantification in rabbit brain and whole blood matrices. Table S2: Precision (CV%) at representative concentration levels, determined by HPLC quantification of AmyP53 in rabbit brain and whole blood matrices. Table S3: HPLC-UV characterization of AmyP53 reference standard (1 mM in water), based on the calibration curve shown in Figure S1 (upper left panel).

## Abbreviations

The following abbreviations are used in this manuscript:

ACN	Acetonitrile
CSF	Cerebrospinal Fluid
ELISA	Enzyme-Linked Immunosorbent Assay
HPLC	High-Performance Liquid Chromatography
LC-MS	Liquid Chromatography–Mass Spectrometry
PBS	Phosphate-Buffered Saline
PF	Preservative Free
