# Supplementary material for: An Optimal Spray Device for the Nose-to-Brain Delivery of AmyP53, an Adaptive Therapeutic Peptide for Alzheimer’s and Parkinson’s Diseases
Source: Pharmaceutics. 2026 Aug 10;18(8):987. doi: 10.3390/pharmaceutics18080987 (PMC13516319; doi:10.3390/pharmaceutics18080987)
Supplement: Supplementary file 1 [file pharmaceutics-18-00987-s001.zip › pharmaceutics-4442551-supplementary.pdf]

Supplementary information

An optimal spray device for the nose-to-brain delivery of AmyP53, an adaptative therapeutic peptide for Alzheimer and Parkinson diseases.

Gonçalo Farias[1], Henri Chahinian[2], Nathalie Hauchard[1], Dominique Brunet[1], Jacques Fantini[2], Nouara Yahi[2], Driss Fantini[3] and Anaïs Aulas[3]*

[1]Aptar Pharma, Le Vaudreuil, France.
[2]Aix-Marseille University, INSERM UA16, Marseille, France.
[3]AmyPore, Septèmes-les-Vallons, France.
*Correspondence: Anaïs Aulas; anais.aulas@amypore.com

Calculation of the percentage of AmyP53 detected in rabbit brain following intranasal administration (representative example at time 10 min post-administration)

The percentage of AmyP53 recovered in the brain following intranasal administration was calculated as follows. A half-brain homogenate (16 mL total volume) was prepared, and half of this volume (8 mL) was extracted at 4°C with 2 volumes of methanol per volume of homogenate (16 mL), yielding a hydroalcoholic phase of 24 mL after centrifugation at 20 000 g at 4°C for 5 minutes. A 50-microliter aliquot of this phase was quantified by ELISA (250 ng, i.e., 0.25 microgram), corresponding to 5 micrograms/mL. This value was extrapolated to the full 24 mL hydroalcoholic phase (120 micrograms), then doubled to account for the whole homogenate (240 micrograms, corresponding to one half-brain), and doubled again to estimate the amount in the whole brain (480 micrograms, i.e., 0.48 mg). Relative to the intranasally administered dose of 12.5 mg, this corresponds to a brain recovery of 3.84% of the administered dose (0.48 mg / 12.5 mg x 100) at this representative time point. The same calculation procedure was applied at each time point of the kinetic study.

Detailed calculations, step by step :

Step 1 - Brain homogenate preparation: A homogenate was prepared from one rabbit half-brain in a total volume of 16 mL.

Step 2 - Methanol extraction: Half of the homogenate (8 mL) was mixed with 2 volumes of methanol per volume of homogenate (2 x 8 mL = 16 mL of methanol), yielding a total hydroalcoholic phase volume of 24 mL (16 mL methanol + 8 mL homogenate).

Step 3 - ELISA quantification: A 50-microliter aliquot of this hydroalcoholic phase was assayed by ELISA, giving a measured amount of 250 ng (0.25 microgram).

Step 4 - Concentration per mL of hydroalcoholic phase: Extrapolating the 50 microliter measurement to 1 mL (factor of 20): 20 x 0.25 microgram = 5 micrograms/mL.

Step 5 - Total amount in the hydroalcoholic phase: Extrapolating to the full 24 mL hydroalcoholic phase volume: 5 x 24 = 120 micrograms.

Step 6 - Total amount in the full homogenate: Since step 2 was performed on half of the original homogenate, this value was doubled to account for the whole homogenate: 120 x 2 = 240 micrograms.

Step 7 - Extrapolation to the whole brain: This value corresponds to the estimated amount in one rabbit half-brain; extrapolating to the whole brain: 2 x 240 micrograms = 480 micrograms (0.48 mg).

Step 8 - Dose administered: The intranasally administered dose of AmyP53 was 12.5 mg.

Step 9 - Percentage of dose recovered in the brain: (0.48 mg / 12.5 mg) x 100 = 3.84%.

Figure S1. Stability of AmyP53 formulation, as assessed by HPLC-UV.

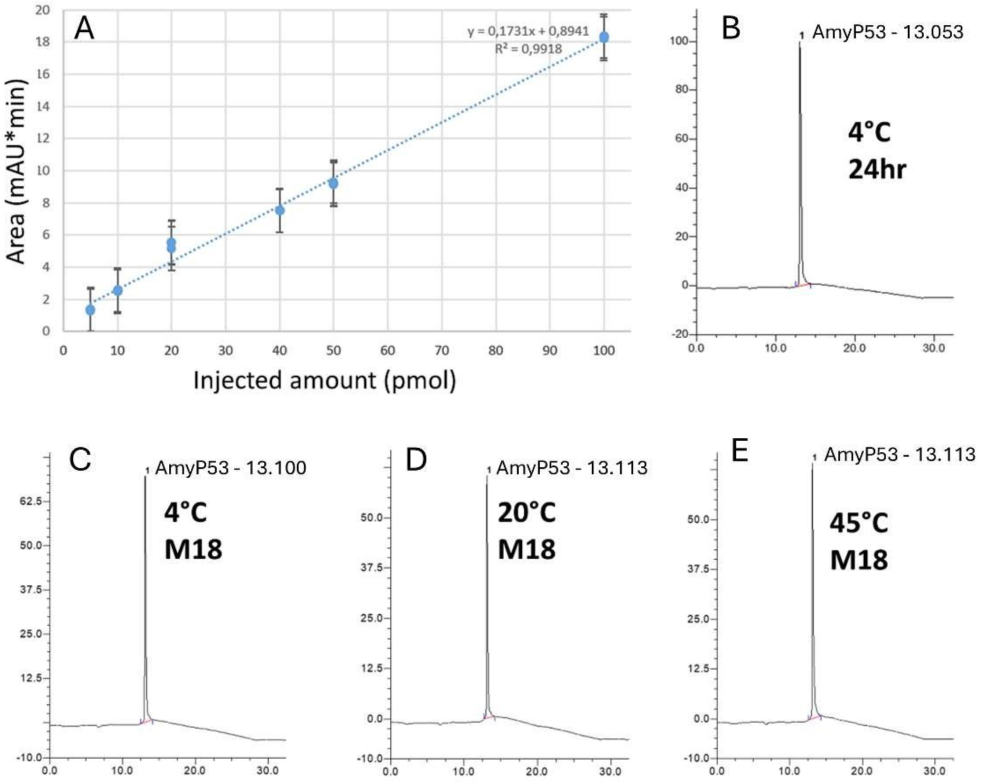

(**A**) Calibration curve of the AmyP53 standard solution (5-100 pmol, y = 0.1731x + 0.8941, R^2 = 0.9918). (**B**) Representative chromatogram after storage at 4°C for 24h (single peak eluting at 13.053 min). (**C-E**) Representative chromatograms after 18 months (M18) of storage at 4°C, 20°C, and 45°C, respectively, demonstrating remarkable stability of aqueous AmyP53 solutions upon prolonged storage at temperatures up to 45°C (single peak eluting at 13.100, 13.113, and 13.113 min, respectively). *Reproduced from Di Scala et al., Int. J. Mol. Sci. 2022, 23(21), 13383, https://doi.org/10.3390/ijms232113383, published under the terms of the Creative Commons Attribution (CC BY) 4.0 license.*

Figure S2. Dosing of AmyP53 by ELISA.

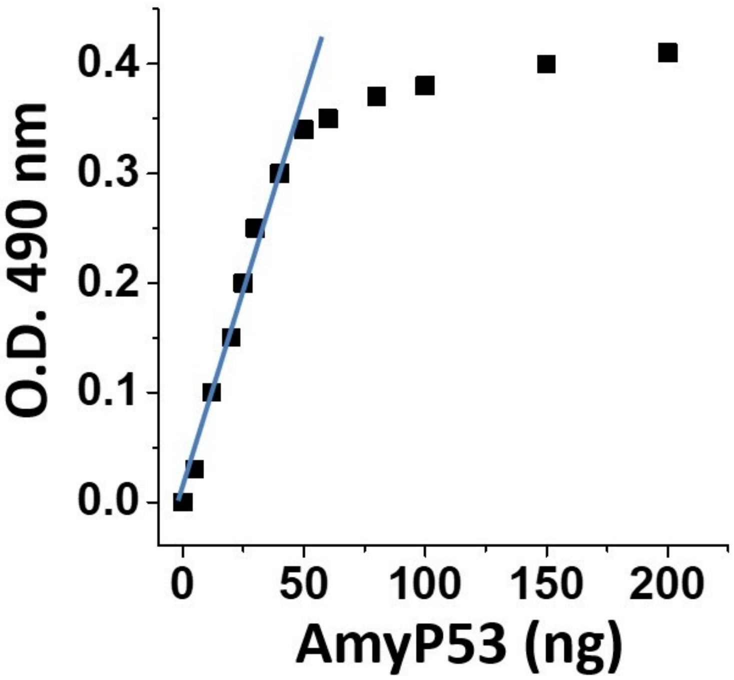

ELISA plates were coated with synthetic AmyP53 in water formulation and incubated with rabbit anti-AmyP53 antibodies, revealed with SIGMAFAST™ OPD substrate (absorbance at 490 nm). The calibration curve showed a linear range between 0-40 ng (R^2 = 0.987), with an estimated limit of detection (LOD) of ~3.9 ng and a lower limit of quantification (LLOQ) of ~11.9 ng. *Modified from El-Battari et al., Int. J. Mol. Sci. 2021, 22(21), 11550, https://doi.org/10.3390/ijms222111550, published under the terms of the Creative Commons Attribution (CC BY) 4.0 license.*

Table S1. Bioanalytical method validation parameters for AmyP53 quantification in rabbit brain and whole blood matrices

Parameter	Rabbit brain	Rabbit whole blood
Calibration range	100-20000 ng/mL	100-20000 ng/mL
Regression model	Weighted (1/X^2) linear	Weighted (1/X^2) linear
Slope	0.000004214	0.000003292
Intercept	-0.0003657	-0.0002490
R^2	0.9668	0.9787
LLOQ	100 ng/mL	100 ng/mL
ULOQ	20000 ng/mL	20000 ng/mL
Specificity	No peptide signal in blank matrix	No peptide signal in blank matrix
LOD	7 ng/mL*	Not formally established below LLOQ**
LLOQ	100 ng/mL	100 ng/mL
ULOQ	20000 ng/mL	20000 ng/mL

*Based on chromatographic peak extrapolation below the validated LLOQ, an approximate detection threshold (without quantification guarantee) of 7 ng/mL was estimated in rabbit brain matrix, consistent with the sensitivity of the reference HPLC-UV method (minimal amount detected: 5 pmol) used for AmyP53 standard characterization. This value should be considered an indicative detection estimate rather than a validated limit of detection, as it falls outside the formally validated calibration range (LLOQ = 100 ng/mL).

**Not formally established below LLOQ in whole blood matrix, as no peak could be detected (see Figure 5D).

Table S2. Precision (CV%) at representative concentration levels, determined by HPLC quantification of AmyP53 in rabbit brain and whole blood matrices.

Concentration (ng/mL)	CV% Rabbit Brain
200	10.8%
1000	16.3%
10000	2.8%
20000	21.2%*

Concentration (ng/mL)	CV% Rabbit blood
200	6.8%
500	13.6%
2500	6.4%
5000	5.8%
20000	2.8%

Precision was assessed as the coefficient of variation (CV%) calculated from duplicate measurements at representative concentration levels spanning the low, mid, and high range of the validated calibration curve (100-20000 ng/mL). All values were within the generally accepted acceptance criteria (CV ≤ 15-20% for QC levels), confirming satisfactory assay reproducibility in both matrices. *One level (20000 ng/mL, brain matrix) showed CV% slightly above the strict 20% threshold, within the wider tolerance sometimes accepted near the upper limit of quantification.

Table S3. HPLC-UV characterization of AmyP53 reference standard (1 mM in water), based on the calibration curve shown in Figure S1 (upper left panel).

**Parameter**	**Value**
Linearity range	5-100 pmol
Regression equation	y = 0.1731x + 0.8941
R^2	0.9918
Minimal amount detected (LOD)	5 pmol
Recovery	>98% (in agreement of the certificate of analysis, batch #P190628-LL735654)
Precision (%RSD, triplicate injections)	Within specified limits (Hubert et al., 1999, Anal. Chim. Acta 391:135-148)
